# Sensory Behaviours and Resting Parasympathetic Functions among Children with and without ADHD

**DOI:** 10.1155/2021/6615836

**Published:** 2021-11-16

**Authors:** Ivan Neil Gomez, Lissa Martha Domondon, Hector WH Tsang, Chetwyn CH Chan, Cynthia YY Lai

**Affiliations:** ^1^Center for Health Research and Movement Science, University of Santo Tomas, Manila, Philippines; ^2^The Graduate School, University of Santo Tomas, Manila, Philippines; ^3^Department of Rehabilitation Sciences, The Hong Kong Polytechnic University, Hung Hom, Kowloon, Hong Kong

## Abstract

Previous studies suggest that parasympathetic functions support sensory behaviours. However, the relationship between sensory behaviours and parasympathetic functions remain inconclusive and inconsistent among children with and without attention-deficit hyperactivity disorder (ADHD). This research aims to examine the sensory behaviours and resting parasympathetic functions among children with and without ADHD. We compared sensory behaviours and baseline parasympathetic functions of 64 participants, with 42 typically developing and 24 ADHD male children aged 7–12 years. Sensory behaviours were evaluated using the sensory profile. Baseline parasympathetic functions were indexed using the normalized unit of heart rate variability high-frequency bands (HF n.u.). Children underwent an experimental protocol consisting of watching a silent cartoon movie while HF n.u. is continuously monitored, within a controlled environment. The results of this research showed significantly lower HF n.u. (*t*(64) = 7.84, *p* < 0.01) and sensory processing total score (*t*(64) = 14.13 = *p* < 0.01) among children with ADHD compared to their typically developing peers. Likewise, a significant moderate positive correlation (*r* = 0.36, *p* < 0.05) was found between the HF n.u. and sensory profile total scores among children with ADHD. Children with ADHD have significantly lower resting state parasympathetic functions compared to their typically developing peers.

## 1. Introduction

Attention-deficit hyperactivity disorder (ADHD) is a childhood neurobehavioural disorder characterized by persistent inattention, hyperactivity, and impulsivity. The prevalence of ADHD has been suggested to reach between 5.29% and 7.1% in children and adolescents and at 3.4% (range 1.2–7.3%) in adults [1]. ADHD can affect children's participation in daily life (i.e., school, social participation, and ADLs) that persists into adulthood [2]. Understanding the underlying neurobiological correlates of ADHD can lead to better evaluation and intervention [3, 4]. Over the past years, several attempts have been made to determine the neurobiological origins of ADHD. Although the exact neuropathology is yet to reach consensus, researchers agree on the neurological basis of ADHD and its symptoms. Specifically, brain imaging studies have implicated the frontostriatal and frontoparietal circuits [5] and the prefrontal-striatal model and primary visual cortex [6]. Nevertheless, procedural requirements have made obtaining good brain imaging data among children with and without ADHD difficult [7, 8]. Measuring the functions of the autonomic nervous system (ANS) has previously been suggested as an alternate method [9].

The ANS, specifically its parasympathetic (PNS) branch, has been linked with ADHD behavioural manifestations [10–12]. Several options on noninvasive, effective, and efficient indexing of ANS functions are available [13]. The PNS, specifically the resting state functions, is responsible for regulation, attention, cognition, and sensory processing [10–12]. Specifically, the high-frequency (HF) bands of heart rate variability (HRV) have been found to represent PNS functions [14]. The PNS resting state functions reflect the psychophysiological flexibility in the regulation of responses [15].

Previous studies suggested that the PNS functions are related to how children with and without ADHD regulate responses to sensory stimuli [11, 16]. Compromised PNS functions may have an effect on how children attain and sustain a relaxed state while interacting with sensory world [3]. Children with ADHD have been shown to have varied difficulties responding to sensory stimulation [17, 18]. Specifically, children with ADHD have been found to be more sensory responsive to sensory stimuli in their everyday environments [19]. There is scant evidence to directly link PNS functions and difficulties in the regulation of response to sensory stimuli [11, 20]. However, maladaptive sensory responsiveness may be a result of difficulty to physiologically regulate the PNS functions in order to achieve homeostasis. It is for these reasons that this study is grounded on.

Children with ADHD have been found to have atypical behavioural and physiological responses to sensory stimuli [21, 22]. One noticeable gap is that the current evidence has been limited mainly to sympathetic functions. Furthermore, evidence on the relationship between behavioural and physiological responses to sensory stimuli has been limited to children with autism spectrum disorder [23–25] and yet to be proven among children with and without ADHD. Methodological variations of instrumentation, experimental procedures, and data analysis [11] may have likely contributed to the current knowledge gap related to the relationship between behavioural and physiological regulation of responses to sensory stimuli among children with and without ADHD.

In this study, we follow salient recommendations suggested by Gomez et al. [11] in using the high-frequency component (HF) of heart rate variability (HRV) to measure parasympathetic functions during an experimental paradigm that reflects a usual experience in daily life. We hypothesised differences between children with and without ADHD on the resting-state parasympathetic functions and the relationship between resting-state parasympathetic functions and sensory processing behaviours.

This research aims to examine the sensory behaviours and resting parasympathetic functions among children with and without ADHD. Specifically, this research sought to: (1) determine differences in sensory behaviours between typically developing children and children with ADHD; (2) determine differences in resting parasympathetic functions between typically developing children and children with ADHD; and (3) determine the relationship between sensory behaviours and resting parasympathetic functions among typically developing children and children with ADHD.

## 2. Methods

A descriptive cross-sectional study will be used in this research. Ethical approval was obtained from the University of Santo Tomas-Graduate School, Ethics Review Board. Before data gathering procedures, assent forms and parental consent forms were distributed to the participants and their respective parents. Whenever discomfort could not be tolerated by the child, the testing procedure was stopped.

### 2.1. Participants

A convenient sampling technique was used to recruit participants in this research. To recruit children with ADHD, invitation letters were sent to therapy centres. Typically developing (TD) children were recruited through partner primary schools of the university. Participants must be males, aged 7–12 years, currently attending a primary school, and have no known history of cardiorespiratory, orthopaedic, or metabolic conditions. TD children have no known history of behavioural, psychological, or developmental disability, while children with ADHD had no comorbid behavioural, psychological, or developmental disability apart from ADHD. A total of 64 participants were recruited in this study, with 42 typically developing (*M* = 8.90 yrs + SD = 1.52) and 24 ADHD (*M* = 9.40 yrs + SD = 1.71) children. Schaaf et al. (2003) previously suggested that at least *n* = 20 for each group (i.e., ADHD and TD) is an adequate number for comparison of differences in PNS functions. For both groups, the mother was the primary caregiver with at least a college-level educational background.

### 2.2. Measures and Instrumentation

Behavioural measures were assessed using the Sensory Profile (SP) and the Participation and Environment Measure for Children and Youth (PEM-CY). The SP is a behavioural checklist that measures how children respond to sensory stimuli as they participate in daily activities [26]. The PEM-CY examines the participation frequency and extent of involvement in the daily activities of children [27]. Both behavioural measures are completed by the child's primary caregiver.

Heart rate variability (HRV) enables this research to look at the physiological activity of the PNS and their interactions across different experimental conditions (i.e., resting baseline). Specifically, the HF band of HRV will be used to represent PNS functions. For this study, a Polar H2 Heart Rate Monitor (Polar, Finland) was used to measure heart rate. The equipment consists of a heart rate monitor that is attached to a chest belt and transmits heart rate signals via an infrared device to a computer notebook. Heart rate monitors such as the Polar H2 allows efficient data acquisition of heart rate variability and is suitable for the paediatric population. There is some evidence that supports the use of Polar heart rate monitors as a valid instrument to measure heart rate variability [28].

### 2.3. Laboratory Paradigm

**Figure 1:**
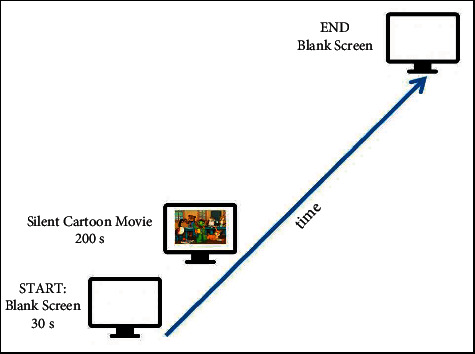
Experimental paradigm.

The experimental paradigm used in this study was adapted from the resting baseline condition of previous similar studies, summarised by Gomez et al. [13]. The experimental paradigm is described to be passive and does not require high cognitive demands from the child. A blank screen was first visually presented on the screen and lasted for thirty seconds. Then, a three-minute resting baseline period was employed to allow sufficient data time points necessary for the analysis of resting PNS functions (HRV- HF). During this time, a neutral silent cartoon movie was shown to the child for 200 seconds. The screen then reverts back to a blank screen which signals the end of the resting baseline period. LabView (National Instruments, USA) was used to design, run, and record time points in the experimental paradigm.  Figure 1 illustrates the experimental paradigm used in this research.

### 2.4. Experimental Procedures

**Figure 2:**
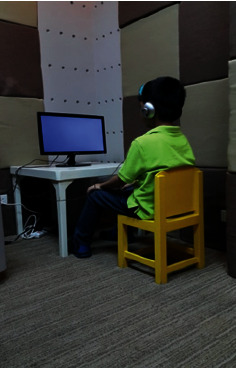
Typical experimental procedures (photo released with permission).

During the day of experimental procedures, participants were greeted by the researcher and led inside the testing room, initially accompanied by the parent where general instructions were given. With the help of the parent, the Polar H2 chest strap was placed just below the chest of the participant. The researcher instructed the child to sit on a comfortable child-sized chair, facing a 19-inch monitor three feet away. A set of over-the-ear closed-back headphones was used to control extraneous noise. During the actual experimental procedures, the room was dimmed to 10 lux (illumination level), temperature set to 23–25°C, humidity level at 60–80%, and background noise level at 40–45 decibels. The researcher was located 60–80 cm away to the left of the participant, keeping interaction minimal. Testing procedures in this research were adapted from a similar previous study [13]. Environmental conditions were recorded before and after the experimental procedures. These conditions were kept constant across all testing.  Figure 2 depicts a typical experimental procedure in this research.

### 2.5. Signal Processing

The aHRV (Nevrokard, Slovenia) software was used to analyse HRV data employing the current standards and guidelines for HRV reporting [14]. The specific procedures for HRV signal processing in this research were adapted from the work of Gomez et al. [13]. Before HRV analysis, tachograms were subjected to succinct preprocessing. Artefacts in HRV seem inevitable in psychophysiological studies. Using the researcher's observation notes, tachograms were subjected to visual analysis, identifying ectopic beats, movement artefacts, and abnormal noise signals. HRV files were then epoched into specific time events in the experimental paradigm. The epoched HRV files were then subjected to the correction of artefacts as employed by the aHRV software. In identifying artefacts for the short-term recordings, the aHRV software compares values to 20% under or over the mean of the preceding 25 beats within the epoched response window (180-second block). Identified noise artefacts are then edited using proper interpolation, keeping as much integrity of the data sample. Data with more than 3% correction from the total normalized HRV data samples were discarded. Frequency domain analysis covered high (HF; 0.15–0.40 Hz)-frequency components in its normalized units. The normalized units of the LF were used as representative of parasympathetic modulation activity.

### 2.6. Data Analysis

Descriptive statistics involving measures of central tendencies and variation are used to describe the salient characteristics of the data gathered. Computed composite scores on the behavioural questionnaires and the HF n.u. were tested for significant differences between children with and without ADHD using an independent *t*-test. Furthermore, a Pearson product-moment correlation coefficient was computed to assess the relationship between behavioural and physiological measures related to the regulation of response to sensory stimuli. SPSS 23.0 was used for all statistical analyses, and the significance level is set at a critical level of 0.05.

## 3. Results

The results of this study are divided into three sections and are summarised in Table 1.

### 3.1. Differences in Sensory Behaviours between Typically Developing Children and Children with ADHD

We compared group differences on the composite scores on sensory processing and participation frequency among children with and without ADHD (*n* = 64). Typically developing children showed significantly higher (*t*(64) = 14.13 = *p* < 0.01) SP scores (*M* = 523 + SD = 49.16) compared to children with ADHD (*M* = 356 + SD = 40.49). No significant differences were found for PEM-CY scores. Both participants engage in a similar frequency of participation in daily activities; however, children with ADHD exhibit significant sensory behaviour problems when facing such activities.

### 3.2. Differences in Resting Parasympathetic Functions between Typically Developing Children and Children with ADHD

Resting-state parasympathetic functions, as represented by the HF n.u., were compared between typically developing (*n* = 42, *M* = 60.16 + SD = 5.97) and ADHD (*n* = 24, *M* = 48.18 + SD = 5.98) children. This research finds significantly lower baseline parasympathetic functions for children with ADHD compared to their typically developing counterparts (*t*(64) = 7.84, *p* < 0.01).

### 3.3. Relationship between Sensory Behaviours and Resting Parasympathetic Functions among Typically Developing Children and Children with ADHD

Behavioural and physiological measures were correlated independently for each group. A significant moderate positive correlation (*n* = 24, *r* = 0.36, *p* < 0.05) was found between the HF n.u. and SP total scores among children with ADHD.

## 4. Discussion

Taken together, the results of this research show that children with and without ADHD participate in daily activities with similar frequency, but those with ADHD experience more problems approaching sensory stimuli with lower parasympathetic functions. More specifically, ADHD children with higher parasympathetic functions exhibit better sensory processing.

The purpose of this study was to examine behavioural and resting parasympathetic functions among typically developing children and children with ADHD. Previous studies found sensory processing difficulties among children with ADHD [29], which corroborates the findings in this study. Dunn [26] suggested that inattention and hyperactivity are outcomes of impaired sensory processing. Likewise, difficulty in sensory processing and modulation has been seen in children with ADHD [18–20]. They may be engaging in similar activities, but their preferences and responses to these activities are different. It has been described previously that sensory processing abilities are the basis for the acquisition of higher cognitive skills such as attention, concentration, and regulation [18]. Whether the deficits in sensory processing are commonly found in ADHD relates to that its behavioural manifestations should be further examined in future studies.

ADHD presents with several behavioural issues, which have been previously linked with an underlying neurological basis. We know from previous research that children with ADHD may have compromised sympathetic functions related to behavioural response to sensory stimuli [11, 21]. The new findings presented in this study suggest that PNS functions may likewise play a role in the regulation of such behavioural responses to sensory stimuli. This research found that the resting-state parasympathetic functions are significantly low in children with ADHD. Lower PNS functioning is related to problems in regulation, attention, cognition, and sensory processing [9, 12, 16, 30] similar to common features of ADHD. Lower resting-state parasympathetic functions in children with ADHD could impact their response to environmental stimuli reflecting inflexible regulatory abilities [15]. We believe that it might be for this reason why children with ADHD have difficulty attaining and sustaining a sense of calm in the face of sensory stimuli. This may explain why children with ADHD have been found to have difficulties in regulating their responses to sensory stimuli, which leads to sensory behavioural problems. This research provides supporting evidence on the association between adaptive sensory behaviour and higher resting-state PNS functions in children with ADHD. Children with lower PNS functions may reflect pathologic neurobehavioral systems responsible for behavioural symptoms [11, 24, 25].

The results from this study provide a novel inquiry on how lowered PNS functions may affect how children with ADHD attain and sustain a calm and focused state that supports behavioural responses to sensory stimuli in daily activities. Our results espouse this idea, and we suggest that PNS functions may be a vital component in the evaluation of atypical sensory behaviours as well as other multifaced behaviours associated with ADHD.

This research is not without any limitations. First, the ADHD sample in this study represents a general clinical population, which may vary in their sensory processing difficulties. Future studies may consider controlling the specific subtype of ADHD and describing their certain sensory issues. Second, while the resting-state parasympathetic functions describe a child's ability to respond, it will be interesting to examine the responsiveness to sensory stimuli by considering a sensory stimulation block to the experimental paradigm. Lastly, the use of a single autonomic measure may not depict a composite physiological response; hence, we recommend describing coexisting sympathetic functions.

## 5. Conclusions

In summary, this research provides evidence supporting significantly lower resting-state parasympathetic functions in children with ADHD compared to their typically developing peers. The results in this research contribute to the growing body of literature supporting the neurophysiological evidence related to the pathology of ADHD. The link between behavioural and physiological measures suggest neurophysiology-based outcome measures and interventions can be considered in addressing symptoms of ADHD. Future studies should address better sampling design, controlling for specific ADHD subtypes, investigate the ability to respond to sensory stimuli, and describe coexisting sympathetic functions.

## Figures and Tables

**Table 1:** Summary table of salient findings.

	TD	ADHD
*Participant characteristics*		
*n* =	42	24
Age (yrs)	8.90 ± 1.52	9.40 ± 1.71
Gender	100% male	100% male
Primary caregiver	95% mother	86% mother
Educational attainment of the primary caregiver	84% college level	78% college level
*Group differences in outcome variables*		
HF n.u.^*∗∗*^	60.16 ± 5.97	48.18 ± 5.98
Sensory profile total score^*∗∗*^	523 ± 49.16	356 ± 40.39
PEM-CY participation frequency	6.39 ± 0.70	6.18 ± 0.71
*Correlation analysis*		
HF n.u. and total sensory profile score	*r* = 0.23	*r* = 0.36^*∗*^
HF n.u. and PEM-CY participation frequency	*r* = 0.28	*r* = 0.27

*Note.* TD = typically developing; HF n.u. = normalized high-frequency band of the heart rate variability; ^*∗*^significant at *p* < 0.05; ^*∗∗*^significant at *p* < 0.01.

## Data Availability

Data are available upon request from the authors.
